# Intra-muscular course of gracilis pedicle in reconstructive surgery – an important anatomic variant

**DOI:** 10.1016/j.jpra.2021.04.002

**Published:** 2021-04-24

**Authors:** Dimitrios Kanakopoulos, Mohamed A. Radhi, Omar Dawood, George Christopoulos, Tomas Tickunas, Christopher Macdonald, Andrew Mellington

**Affiliations:** a Queen Victoria NHS Foundation Trust, Holtye Road, East Grinstead, RH19 3DZ, United Kingdom; b University Hospitals Sussex NHS Foundation Trust, Eastern Road, Brighton, BN2 5BE, United Kingdom

## Introduction

The gracilis muscle or musculocutaneous flap is a commonly used flap in reconstructive surgery.^1^ Its vascular pedicle originates from the medial circumflex femoral artery, branch of the profunda femoris artery or occasionally originates for the profunda femoris itself and can be reliably found in the septum between adductor magnus and adductor longus muscle.^2^ Its harvest is relatively straightforward for the reconstructive surgeon and its loss is very rarely of any functional significance to the patient, such as in professional athletes.^3^ Its applications vary from pedicled to free and has a broad range of indications, such as regional reconstruction of lower abdomen, pubis, groin, perineum, ischium, including functional anal sphincter and vaginal reconstruction, and distant reconstruction for head & neck, including functional reconstruction for facial reanimation, as well as for upper & lower extremity, including functional reconstruction for muscle loss.^4^, ^5^, ^6^, ^7^, ^8^, ^9^, ^10^, ^11^, ^12^, ^13^

**Figure 1:**
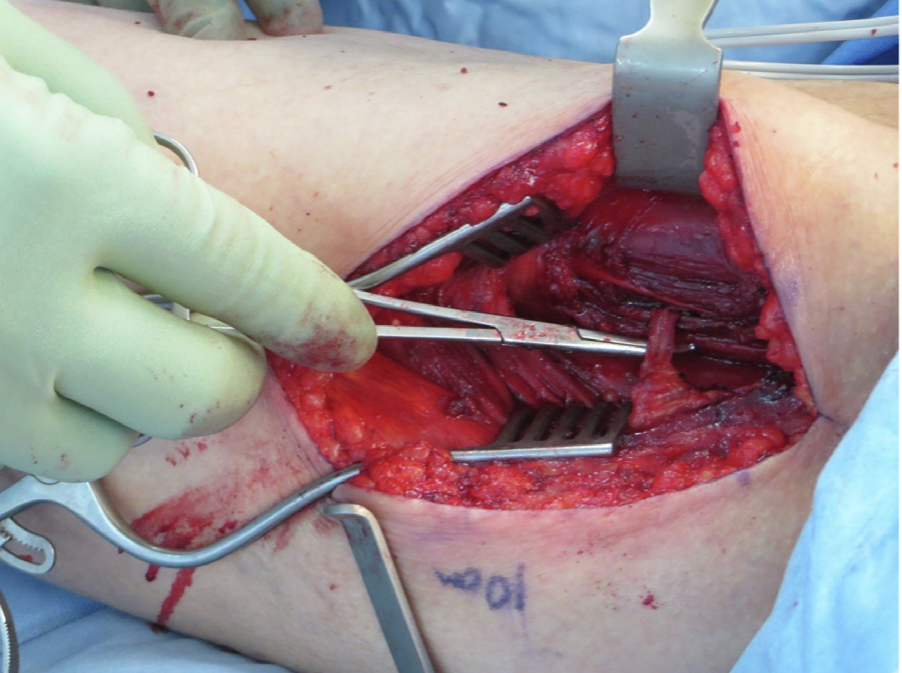
Demonstration of the pedicle to gracilis muscle dissected through muscle substance of adductor magnus muscle – total intramuscular course.

Here we present two individual cases of unilateral intramuscular course of the gracilis pedicle (Figure 1), that we e ncountered in two separate patients requiring soft tissue reconstruction for open distal tibia/fibula fracture following trauma. In both cases, free gracilis muscle transfer was deemed appropriate means of reconstruction of distal 1/3 of the leg soft tissue defect, where local reconstructive options are very limited or absent.

## Case 1

A 76 y.o. female with a left open distal tibia/fibula fracture following trauma sustained after a fall from stairs, had reconstruction with a contralateral free gracilis muscle flap. During harvest, the course of the pedicle to gracilis muscle was found to be entirely intramuscular through adductor longus, requiring a perforator flap type of dissection to free up the 7 cm long pedicle. This practice added significant difficulty in raising the flap and increased the length of time of the overall procedure. Regrettably, this gracilis muscle flap subsequently failed 4 days post op. A decision to re-do the reconstruction with an ipsilateral free gracilis muscle flap was made, giving the team the opportunity to compare the anatomic variation of the pedicle to gracilis with the opposite side. On this occasion, the course of the pedicle was the one commonly encountered, that was in the intermuscular septum between adductor magnus and adductor longus muscle, ensuring a speedy and uneventful dissection. The outcome was successful.

## Case 2

A 74 y.o. female with a left open distal tibia/fibula fracture following trauma sustained after a fall from own height, had reconstruction with a contralateral free gracilis muscle. Again, during flap harvest the gracilis pedicle was found to have an intramuscular course through adductor longus, however, this time the course was partly intramuscular – 3 cm of pedicle length. Although the dissection was not as tedious as in Case 1, it still added in surgical effort and operative time. The outcome was successful.

## Discussion

The gracilis muscle is a Mathes & Nahai type II muscle. It originates at the ischiopubic ramus and inserts on to the upper medial tibia below the medial condyle. Its nerve supply comes from the anterior branch of the obturator nerve. The vascular pedicle that supplies the muscle takes off from the medial circumflex femoral artery, a branch of the profunda femoris artery, runs downwards between the adductor longus and magnus and enters the lateral border of the muscle 8–12 cm below the pubic tubercle at the junction of the proximal and middle third of its length, its diameter being 1.5–2 mm at its origin. As it enters the muscle, the pedicle divides into three to six musculocutaneous perforators that will go on to supply the skin overlying the upper one third of the gracilis muscle. Venous drainage is noted to be through paired venae commitantes, which often become one large vein just before joining the profunda femoris vein.^15^

Distal to the main vascular pedicle usually lies multiple (one-10.4%, two-64.6% or three 25%) minor pedicles. However, a definitive minor pedicle (85.4%) is present proximal to the main pedicle.^4^ The middle and distal third of the muscle is supplied most commonly by two minor branches from the superficial femoral artery.^14^ Both musculocutaneous and septocutanous branches of the minor pedicles reach the skin overlying the lower third of the gracilis muscle.

Variations of the main vascular pedicle have been previously described in animal studies revealed six distinct origins of the dominant arterial pedicle and four venous pedicle variants.^16^

In 2004, Lasso et al. reported a main vascular pedicle of the gracilis muscle penetrating the fascia of the adductor longus muscle where 3 cm of the pedicle had to be dissected in the same way as for perforator flaps before leaving the intramuscular compartment.^17^

Peek et al. (2009)’s cadaveric study of 43 gracilis perforator flaps. A doubled main vascular pedicle was seen in nine of 43 cases (21%). In seven of these (16%), there was a common origin from the profunda femoris; in two cases (5%), both pedicles had their own origin from the profunda femoris.^18^

Morritt et al. (2014), in a letter to the editor of JPRAS, described a total intramuscular course of a dominant vascular pedicle unilaterally in a 28-year-old woman that underwent unilateral skin sparing mastectomy for invasive ductal carcinoma and immediate reconstruction with bilateral TMG flap. Where the entire course of the pedicle (7 cm), was dissected free from the adductor longus, back to its origin at the profunda femoris.^19^

Natoli et al. (2015) studied 36 TUG flaps performed in 24 patients and reported six aberrant vascular pedicles (17%), four out of which displayed a double main pedicle (11%) and two displayed a split proximal pedicle which originated from the medial circumflex femoral vessels (5.5%). Three out of the four cases that displayed a double main pedicle were confirmed to have 1 pedicle coming from the medial circumflex vessels and the other originating from the deep femoral vessels. In the fourth case of a double main pedicle, both pedicles originated from the medial circumflex vessels.^20^

## Conclusion

Although gracilis muscle flap planning and execution has been established as a straightforward one, our two cases presented here, in conjunction with previous reports in the literature observing encounters of intramuscular course of pedicle to gracilis muscle, confirm that this anatomic variant is frequent and must be included and clearly highlighted in international bibliography. It may, therefore, be prudent for the reconstructive surgical community to consider this anatomic variation when planning to harvest a free gracilis muscle flap, and to proceed with caution during pedicle dissection. That would avoid inadvertent ligation of the dominant pedicle supplying the flap, if erroneously perceived for a non-dominant perforator, assuming an intramuscular course of the pedicle to gracilis is non-existent or extremely uncommon.

## Funding

This paper was exclusively self-funded, and the authors have no financial interest to declare in relation to the content of this article.

## Declaration of Competing Interest

The authors declare no conflicts of interests.
